# The clinical significance of renal resistance index (RRI) and renal oxygen saturation (RrSO2) in critically ill children with AKI: a prospective cohort study

**DOI:** 10.1186/s12887-023-03941-2

**Published:** 2023-05-06

**Authors:** Huili Shen, Weilan Na, Yichu Li, Dong Qu

**Affiliations:** 1 Pediatric Intensive Care Unit, Children’s Hospital of Fudan University, National Children’s Medical Center, Shanghai, China; 2 Pediatric Critical Medicine Department, Children’s Hospital of Capital Institute of Pediatric, Beijing, China

**Keywords:** Acute kidney injury (AKI), Renal resistance index (RRI), Renal oxygen saturation (RrSO2)

## Abstract

**Objective:**

The purpose of this study was to look into the clinical significance of the renal resistance index (RRI) and renal oxygen saturation (RrSO2) in predicting the development of acute kidney injury (AKI) in critically ill children. A new non-invasive method for the early detection and prediction of AKI needs to develop.

**Methods:**

Patients admitted to the pediatric intensive care unit (PICU) affiliated with the capital institute of pediatrics from December 2020 to March 2021 were enrolled consecutively. Data of clinical information, renal Doppler ultrasound, RrSO2, and hemodynamic index within 24 h of admission were prospectively collected. Patients were divided into two groups: the study group was AKI occurred within 72 h, while the control group did not. SPSS (version 25.0) was used to analyze the data, and *P* < 0.05 was considered a statistical difference.

**Results:**

1) A total of 66 patients were included in this study, and the incidence of AKI was 19.70% (13/66). The presence of risk factors (shock, tumor, severe infection) increased the incidence of AKI by three times. 2) Univariate analysis showed significant differences in length of hospitalization, white blood cells (WBC), C-reactive protein (CRP), renal resistance index (RRI), and ejection fraction (EF) between the study and control groups (*P* < 0.05). There were no significant differences in renal perfusion semi-quantitative score (*P* = 0.053), pulsatility index (*P* = 0.051), pediatric critical illness score (PCIS), and peripheral vascular resistance index (*P* > 0.05). 3) Receiver operating characteristic (ROC) curve showed that if RRI > 0.635, the sensitivity, specificity, and AUC for predicting AKI were 0.889, 0.552, and 0.751, respectively; if RrSO2 < 43.95%, the values were 0.615, 0.719 and 0.609, respectively; if RRI and RrSO2 were united, they were 0.889, 0.552, and 0.766, respectively.

**Conclusions:**

The incidence of AKI is high in PICU patients. And infection, RRI, and EF are risk factors for AKI in PICU patients. RRI and RrSO2 have certain clinical significance in the early prediction of AKI and may provide a new non-invasive method for early diagnosis and prediction of AKI.

## Background

Acute kidney injury (AKI) is characterized by a rapid decline in kidney function caused by various factors (shock, sepsis, surgery). The clinical manifestations of AKI are a rapid rise in serum creatinine (SCr) accompanied or not accompanied by decreased urine volume, azotemia, and water/acid-base electrolyte imbalance. A global epidemiological study of AKI [1] showed that the incidence of AKI in intensive care unit (ICU) was about 30%, and the mortality rate was about 3.4%. In PICU, the proportion of septic shock with AKI is as high as 41–73% [2], and 35.7% of children develop AKI STAGE II or III AKI [3]. Septic shock with AKI resulted in death or disability in 64% of children.

At the moment, AKI is primarily diagnosed based on changes in urine volume and SCr. Due to the strong reserve function of the kidney, both of them lag behind the injury and recovery of renal function [4], which are not conducive to the early recognition of AKI. Some non-invasive, effective, bedside and convenient diagnostic indicators are expected to be discovered constantly.

Doppler ultrasound is a non-invasive, rapid, repeatable, and inexpensive examination tool increasingly used in diagnosing and treating critical patients. Renal resistance index (RRI) based on Doppler ultrasound is the ultrasonic signal of interlobar artery blood flow at the Renal cortical medullary junction obtained [5], which is now considered to reflect renal perfusion and vessels [6, 7]. Moreover, RRI had a predictive effect on AKI diagnosis and can reasonably evaluate kidney structure and function.

Near-infrared spectroscopy (NIRS) [8] is a non-invasive bedside tissue oxygen saturation monitoring instrument that can reflect the oxygen supply and demand status of tissues and organs about 4–6 cm beneath the skin [9, 10]. The value of NIRS can be obtained instantaneously and is not affected by low temperature, hypoperfusion, and arterial contraction. Patients with cardiac arrests can also use it. Studies showed that NIRS could accurately detect changes in tissue oxygen. The subcutaneous tissue of children is thin, and the kidney is a retroperitoneal organ close to the skin; kidney tissue oxygen saturation can be monitored.

Existing research indicates that AKI is a common microcirculation disorder. RRI and RrSO2 can theoretically reflect the pathophysiological mechanism of AKI. RRI combined with RrSO2 may theoretically be an effective indicator for clinical prediction and evaluation of AKI, but no relevant scientific studies have been conducted to date. In this paper, RRI and RrSO2 of patients were obtained prospectively within 24 h of admission, and the clinical effects in predicting AKI occurrence. We aimed to provide a non-invasive and convenient bedside method for the early diagnosis of AKI.

## Methods

### Clinical data

All patients admitted to the PICU of our hospital from December 2020 to March 2021 were enrolled. Within 24 h of admission, clinical data, renal Doppler ultrasound data, and NIRS data were collected from patients. Patients were divided into study and control groups according to whether AKI occurred in the first 72 h or not, respectively.

### Inclusion criteria and exclusion criteria

#### Inclusion criteria

(1) Age from 29 days to 18 years; (2) The diagnostic criteria for AKI were according to KDIGO grading criteria, and PCIS was based on pediatric critical case score; (3) Vasoactive drug score (VIS) = dopamine+dobutamine+ 10 × milinone+ 100× epinephrine + 100× norepinephrine(ug/kg/min) -.

#### Exclusion criteria

(1) Age ≤ 28 days or age ≥ 18 years old; (2) Kidney distance measured by ultrasound exceeds NIRS detection range; (3) Patients with chronic kidney dysfunction, underwent kidney surgery or renal vascular abnormalities, renal tumor, renal mass, renal dilatation, hydronephrosis, etc.; (4) Length of the stay less than 72 h; (5) Patient or parental refusal to participate in the study.

### Research methods

#### All the enrolled patients were treated according to relevant diagnosis and treatment standards

All the data were collected, which include (1) demographic information: age, sex, height, weight, and vital signs; (2) laboratory test results; (3) bedside ultrasound values: renal length and width, renal blood perfusion semi-quantitative score, RRI, renal Pulpability index (PI); (4) hemodynamic index; (6) prognostic data of patients: PCIS, VIS, fluid intake and hospital length of stay, the incidence of AKI 72 h after admission. RRI = (systolic peak velocity–end-diastolic blood flow rate)/systolic peak velocity. PI = (peak systolic tachycardia–end diastolic blood flow rate)/[peak systolic tachycardia+end diastolic blood flow rate)/2].

**Fig. 1:**
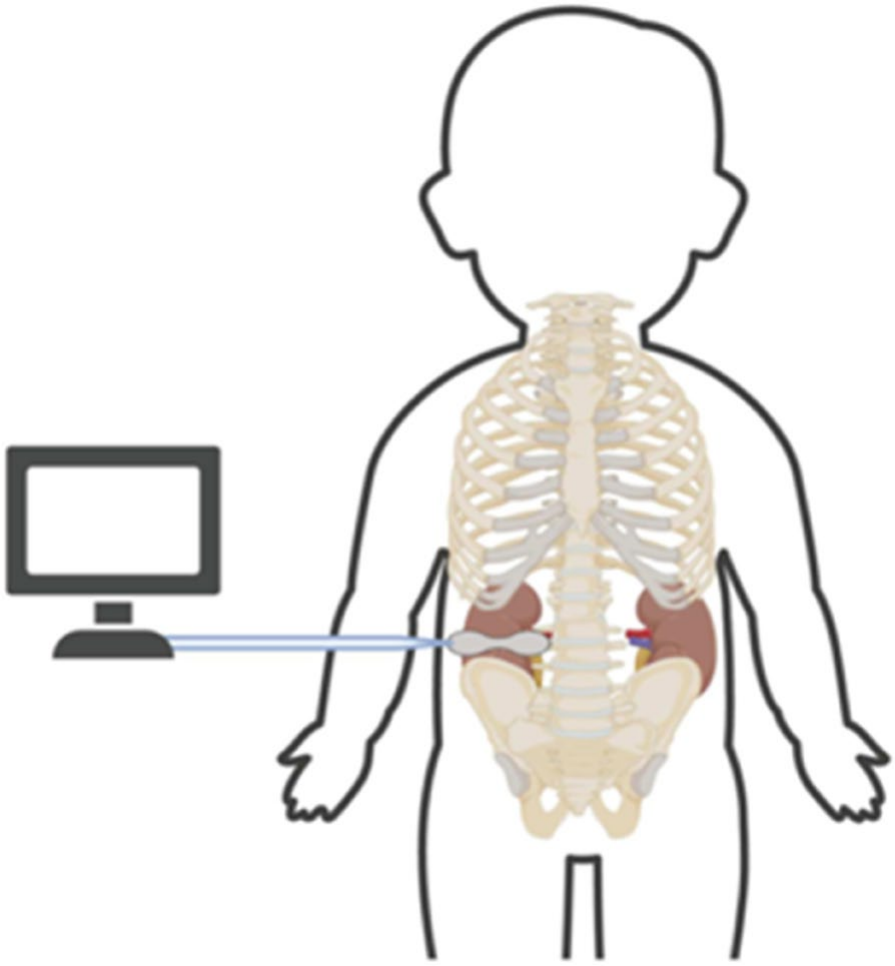
NIRS usage and probe placement

According to the anatomical point (the renal hilum is located at the rib ridge angle) and the retest position of the renal ultrasound positioning, the probe was placed at the point on the surface of the kidney and kept attached without a light source around it. The patient’s information was considered as input, and then the measurement started. After making a stable measurement for 5 min, RrSO2 was obtained. Figure 1 presents the specific measurement method.

USCOM instrument (USCOM, Austria, non-invasive Doppler hemodynamic instrument) was used for getting hemodynamic indicators.

### Statistical methods

For statistical analysis, SPSS software (version 25.0) was used. The central tendency of a normal distribution or near normal distribution was expressed by mean ± standard deviation (Mean ± SD). The quantitative data were tested using an independent sample t-test, and the qualitative data were tested using *X*^2^ test. The median (interquartile spacing) [M(IQR)] was used to represent the central tendency of the non-normal distribution using the rank-sum test (Mann-Whitney U test).

Logistic regression analysis was used for multivariate analysis. After the regression curve was obtained, RRI, RrSO2, and combined application RRI and RrSO2 were used to plot Receiver operating characteristic (ROC) curves. A statistically significant difference was defined as *P* < 0.05.

## Results

### Incidence of AKI and risk factors in PICU patients

A total of 66 patients were included, encompassing 39 males and 27 females, with a male to female ratio of 1.44:1. AKI occurred in 13 (19.7%) patients within 72 h of admission. The incidence of AKI was 19.70% (13/66), with AKI stage I occurring on 5/13 (38.46%), AKI stage II appearing on 4/13(30.77%), and AKI stage III on 4/13(30.77%). Table 1 lists the specific risk factors for AKI that were present in 24 of the 66 patients. Among the 24 patients with risk factors, 8/24 (33.3%) had AKI. AKI occurred in 5/24 (11.9%) patients without identifiable risk factors. The incidence of AKI is approximately three times higher when risk factors are present.

**Table 1:** Analysis of risk factors in 24 patients

Risk factors	Count (%)
Shock (including septic shock)	7(29.2%)
Surgery	4(16.7%)
Sepsis (septic shock not including)	4(16.7%)
Tumor or cancer	10(41.7%)
Chemotherapeutic drugs	4(16.7%)
Others: hyperammonemia	1(4.2%)

A patient may have multiple risk factors

### Univariate analysis of AKI patients

In order to reduce the influence of age, gender, and other demographic baselines, SPSS was used to match the patients: Study group: control group = 1:2.5 (matching index: Age, sex, weight, and height). After matching, we had 13 patients in the study group and 33 in the control group.

**Fig. 2:**
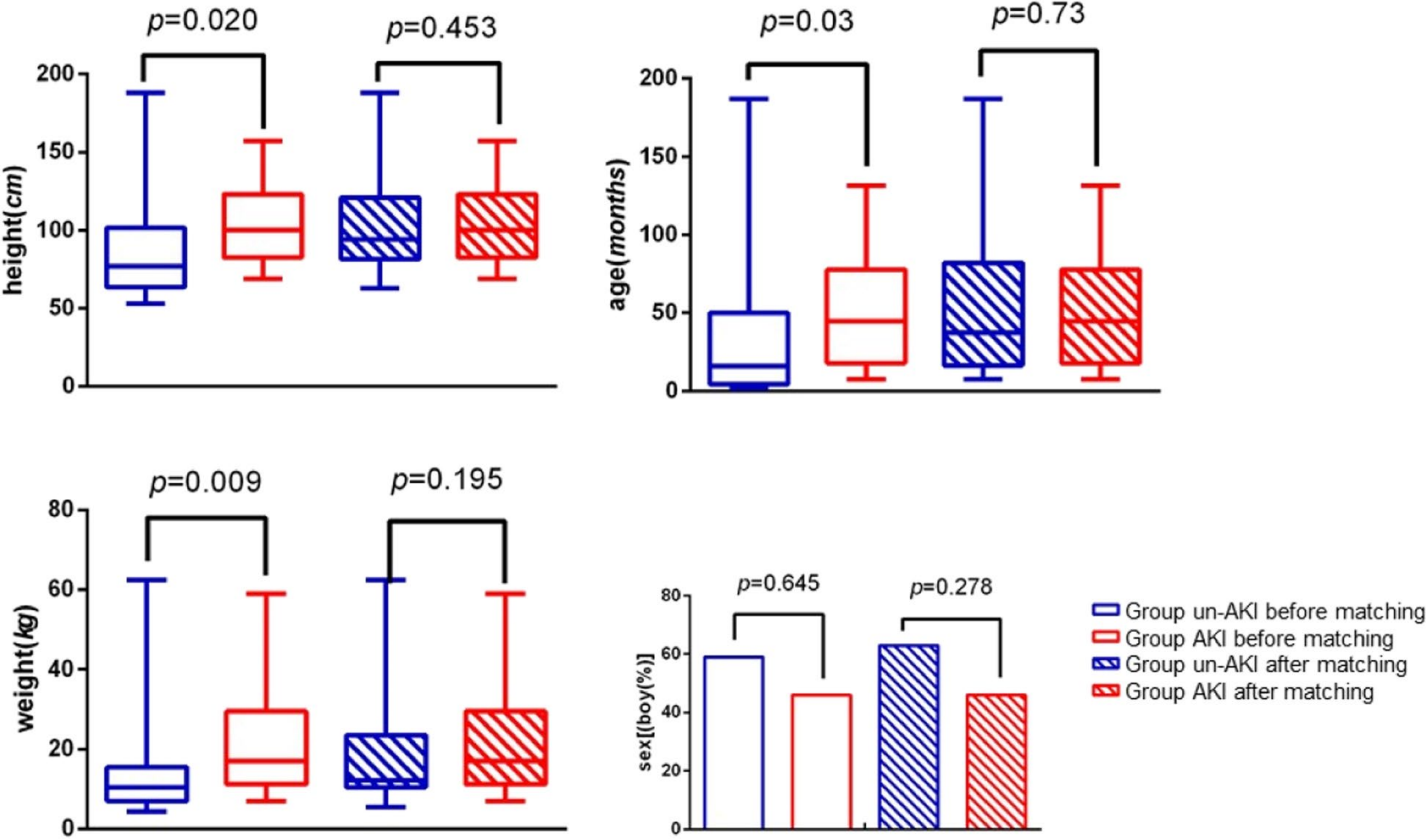
Population characteristics before and after matching

As shown in Fig. 2, no statistical difference was found in baseline values of age, gender, weight, and height between the two groups (*P* > 0.05).

Univariate analysis revealed statistical differences between the two groups in length of hospital stay, WBC, CRP, RI, and Ejection fraction (EF) (*P* < 0.05). Although no statistical differences in a semi-quantitative score of renal perfusion and PI were found, the *P*-value of the two groups was at the critical value of 0.05. The former is 0.053 and the latter 0.051. Table 2 shows that other indicators, such as many risk factors, PCIS value, and peripheral blood flow dynamics parameters, showed no statistical difference (*P* > 0.05).

**Table 2:** Univariate analysis of AKI patients

Demographic	Whole cohort (*n* = 46)	AKI (*n* = 13)	No AKI (*n* = 33)	Statistic	*P* value
Sex [male, count (%)]	27(58.70)	6(46.20)	21(63.60)	1.176	.278
Age (*Mean ± SD,* M)	49.95 ± 38.35	53.11 ± 39.85	48.71 ± 38.30	−.341	.730
Hospital stays [*M(IQR)*, d]	6.00(4.25,9.00)	5.00(4.00,5.75)	8.00(5.00,10.75)	−2.122	.035
Risk factors [count (%)]	20(43.48)	8(61.50)	12(36.40)	2.405	.121
P/F (*Mean ± SD,* score)	242.23 ± 101.77	254.68 ± 135.56	236.37 ± 85.92	−.412	.684
PCIS (*Mean ± SD,* score)	89.30 ± 8.74	88.46 ± 8.69	89.64 ± 8.87	.407	.686
MAP (*Mean ± SD,* mmHg)	75.09 ± 14.52	77.08 ± 15.42	74.30 ± 14.32	−.579	.565
WBC[*M(IQR)*, ×10^9^/L]	9.20(4.40,11.64)	11.40(9.74,17.21)	6.47(3.60,10.18)	−2.818	.005
Hb (*Mean ± SD,* g/L)	98.00 ± 23.52	89.62 ± 30.01	101.30 ± 20.01	1.541	.131
CRP[*M(IQR)*, mg/L]	5.66(1.23,19.50)	12.73(4.98,40.11)	4.25(0.60,15.01)	−2.038	.042
Lac[*M(IQR)*, mmol/L]	1.50(1.08,2.33)	1.50(1.00,3.22)	1.50(1.05,2.05)	−.122	.903
PCT[*M(IQR)*, ng/ml]	0.49(0.17,2.16)	0.54(0.23,11.82)	0.45(0.15,2.04)	−1.122	.262
Cr[*M(IQR)*, μmol/L]	27.20(19.98,32.15)	32.30(26.35,38.45)	24.50(19.85,29.80)	−1.752	.104
RI(*Mean ± SD*)	0.65 ± 0.11	0.71 ± 0.09	0.63 ± 0.10	−2.374	.023
Semi-quantitative score of renal perfusion [*M(IQR)*, score]	3.00(1.25,3.00)	1.50(0.25,3.00)	3.00(2.00,3.00)	7.675	.053
RrSO2 (*Mean ± SD*, %)	46.03 ± 18.45	40.43 ± 20.52	48.31 ± 17.36	1.309	.197
Kidney length (*Mean ± SD*, cm)	6.62 ± 0.97	6.84 ± 1.25	6.51 ± 0.82	−1.015	.316
Kidney width (*Mean ± SD*, cm)	3.36 ± 0.51	3.42 ± 0.56	3.33 ± 0.50	−.532	0.50
PI (*Mean ± SD*)	1.08 ± 0.29	1.24 ± 0.33	1.02 ± 0.26	−2.017	.051
EF[*M(IQR)*, %]	70.00(63.00,72.00)	60.50(48.50,69.75)	71.00(66.00,72.50)	−2.469	.014
VIS[*M(IQR)*, score]	0.00(0.00,0.00)	0.00(0.00,2.50)	0.00(0.00,0.00)	−.430	.667
Intake and output volume (*Mean ± SD*, ml/d)	388.92 ± 544.33	352.62 ± 485.49	403.23 ± 572.30	.281	.780
SVI (*Mean ± SD*, ml/m^2^)	34.35 ± 11.63	34.08 ± 10.63	34.45 ± 12.158	.098	.922
CI (*Mean ± SD*, L/min/m^2^)	4.37 ± 1.45	4.22 ± 1.31	4.43 ± 1.52	.448	.657
SVRI *(Mean ± SD*, ds·m^2^/cm^5^*)*	1477.02 ± 754.69	1461.08 ± 674.512	1483.30 ± 793.84	.089	.930
SVV (*Mean ± SD*, %)	43.48 ± 23.95	34.62 ± 21.45	46.97 ± 24.29	1.602	.116
DO2[*M(IQR)*, ml/min]	328.00(233.00,524.00)	328.00(200,627.50)	343.00(233.00,502.00)	−.180	.857

*PCIS* Pediatric critical illness scoring, *MAP* Mean arterial pressure, *WBC* White blood cell, *HB* Hemoglobin, *CRP* C-reactive protein, *Lac* Lactate, *PCT* Procalcitonin, *Cr* Creatinine, *RI* Renal index, *PI* Pulpability index, *RrSO2* renal regional tissue oxygen saturation, *EF* Ejection fraction, *SV* Stroke volume, *SVI* Stroke volume index, *CI* Cardiac index, *SVRI* Systemic vascular resistance index, *SVV* Stroke volume variability, *DO2* Oxygen delivery

### Multifactor analysis of AKI patients

Logistic regression was used to examine the indexes that showed statistical differences in univariate analysis: RRI, WBC, CRP, and EF. Since the length of hospitalization is a prognostic factor and is not an independent risk factor for AKI in clinical interpretation, the length of hospitalization was not included in the logistic regression analysis. The results showed that none of the above factors were independent risk factors for AKI in PICU patients. The independent risk factors for ICU admission should be investigated further.

### Predictive value of RI, RrSO2, and RRI combined with RrSO2 for AKI occurrence

**Fig. 3:**
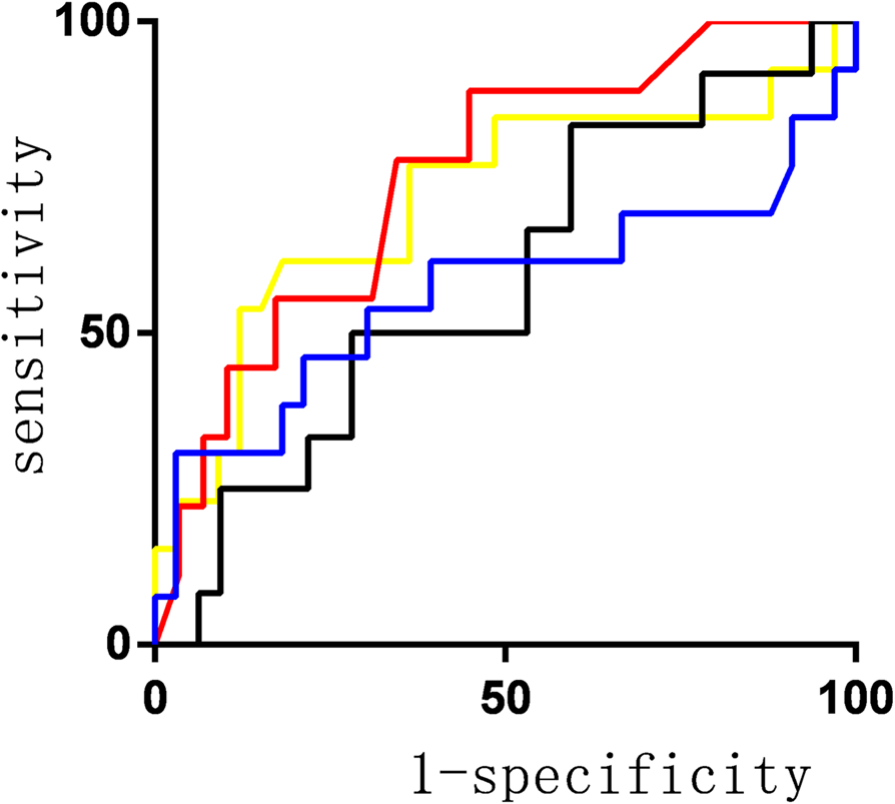
ROC curve for RI, RrSO2 and combined

ROC curve showed that the sensitivity, specificity, and area under the curve (AUC) for predicting AKI in ICU patients with RI > 0.635 were 0.889, 0.552, and 0.751, respectively, while those data with RrSO2 < 43.95% were 0.615, 0.719 and 0.609 respectively. The sensitivity, specificity, and AUC were 0.889, 0.552, and 0.766 when they were combined, as shown in Fig. 3 and Table 3.

**Table 3:** Predictive value of RI, RrSO2 and RRI combined with RrSO2 for AKI occurrence

Item	Sensitivity	Specificity	AUC	*P*	95%*CI*	
					upper	lower
RI > 0.635	.889	.552	.751	.025	.574	.928
RI combined RrSO2	.889	.552	.766	.017	.587	.946
RrSO2 < 43.95%	.615	.719	.609	.328	.403	.815
Cr > 27.1 μmol/L	.769	.424	.714	.025	.530	.899

Renal index

*ROC* Receiver operating characteristic, *AUC* Area under curve, *CI* Confidence interval, *RrSO2* renal regional tissue oxygen saturation

### Renal ultrasonography and dynamic changes of RrSO2 in patients with shock patients

As previously stated, shock is a risk factor for AKI. Renal Doppler ultrasound and renal NIRS values of patients with shock were continuously monitored to understand better the predictive value of RrSO2 combined with RRI in AKI.

**Fig. 4:**
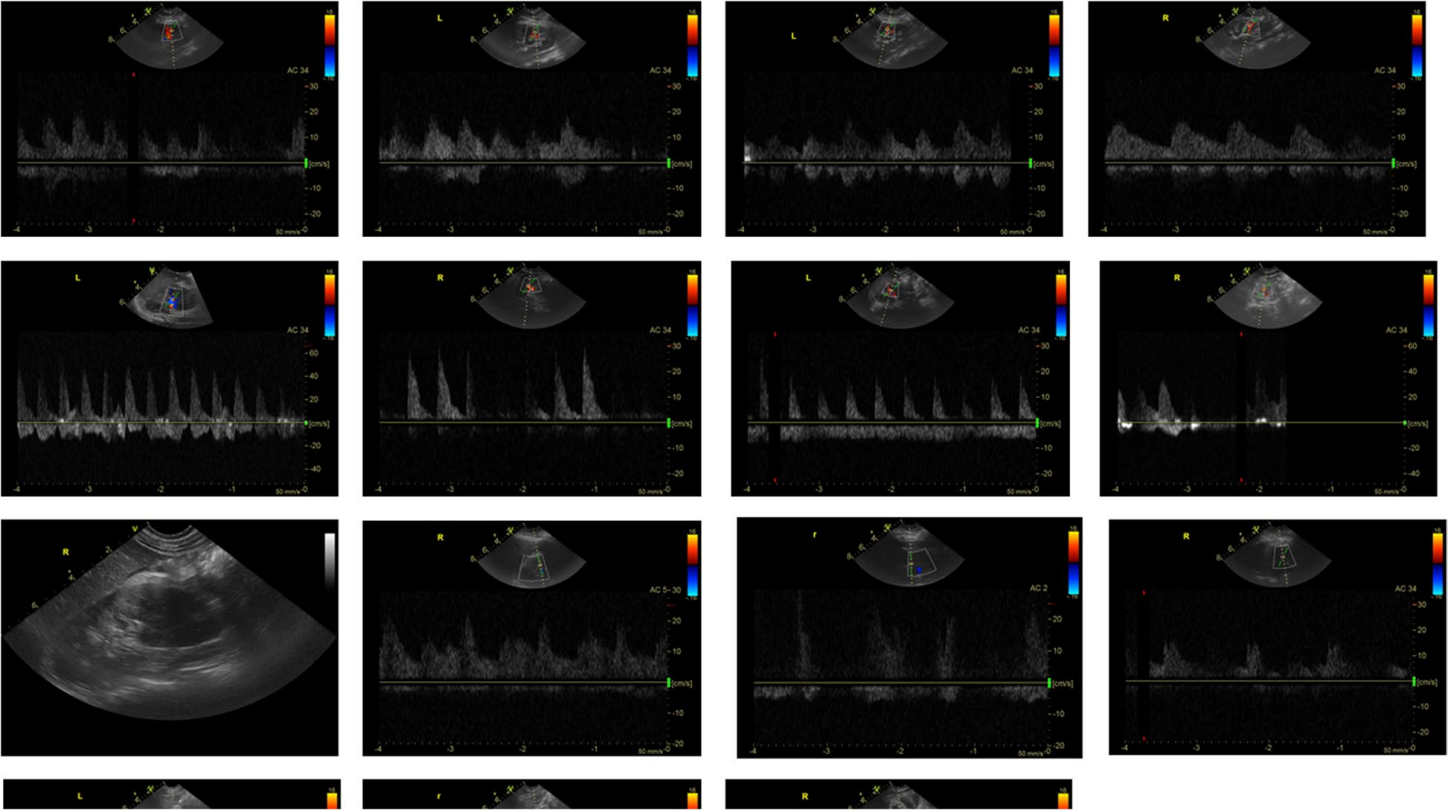
Dynamic ultrasound changes of shock patients. Note: From top to bottom, each line represents a patient, and from left to right each line represents the ultrasound changes of the patient from admission to the fourth day respectively

**Fig. 5:**
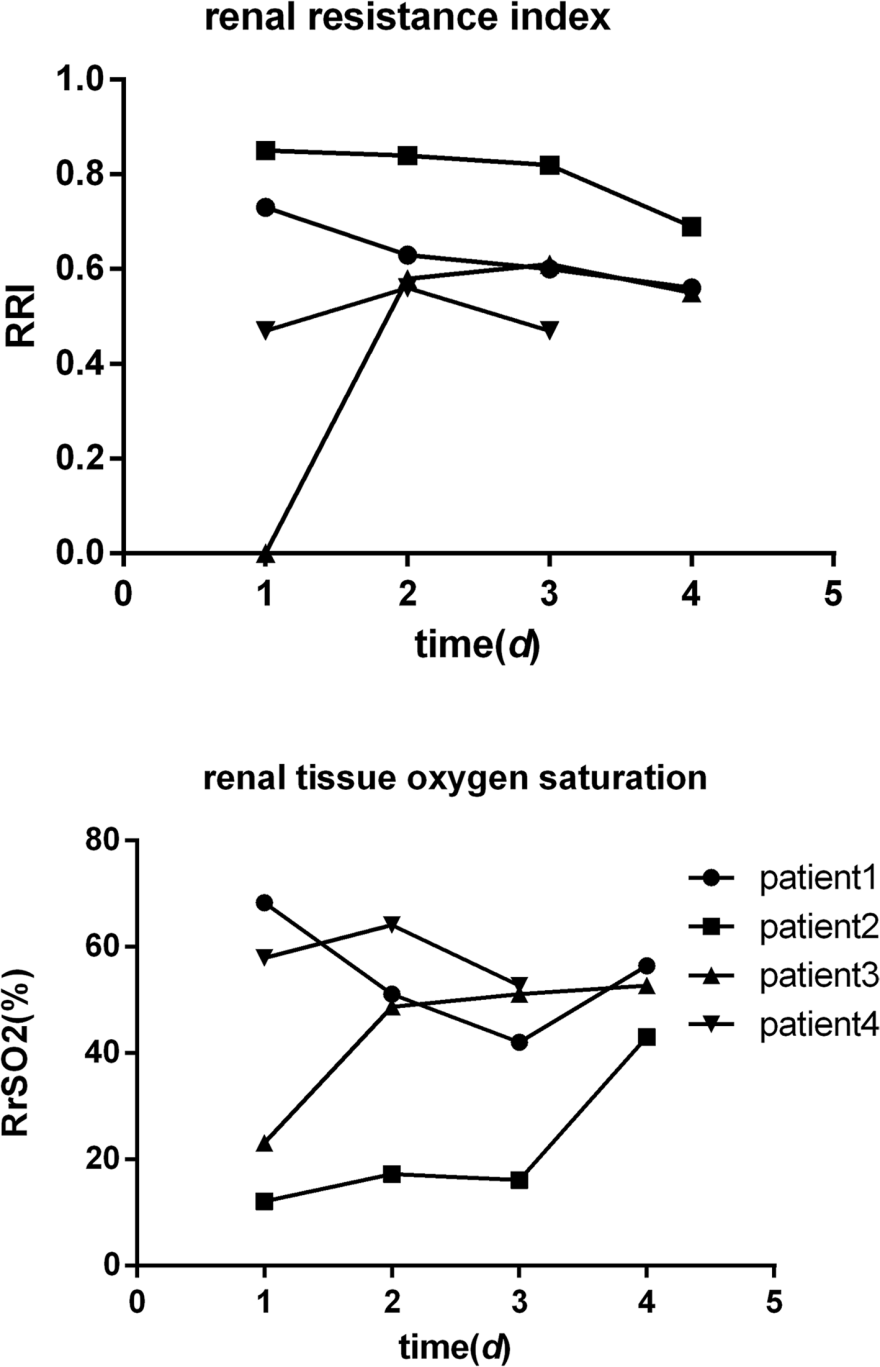
Changes in RrSO2 and RRI at 4 patients

All the following shock patients recovered and were discharged. The results showed that RI gradually decreased and RrSO2 gradually increased with the improvement of patients’ condition. Figures 4 and 5 depict the specific findings of the study.

## Discussion

AKI is a common disease in intensive care medicine. Epidemiological survey results show that the overall incidence of AKI in China is 11.6% [11], and the incidence of AKI in ICU patients globally is 26.9% [1]. This study found that the incidence of AKI in PICU is 19.70% (13/66), roughly consistent with previous studies. Various factors can cause AKI, and common clinical risk factors include shock, sepsis, tumor, etc. This study found that the existence of risk factors can increase the incidence of AKI by about three times, and patients with AKI will have more than two times of risk factors (see Table 4). Kaddourah [1] et al. found that septic shock was the main risk factor for AKI, which was consistent with this study. Furthermore, this study found that tumors, especially tumor cytolysis, would increase the incidence of AKI in patients, which is considered related to increased metabolic pressure in the kidney after tumor cell lysis. Thus, patients with tumors, especially those with tumor cytolysis, should be paid attention to in the clinic.

**Table 4:** Logistic regression analysis of risk factors for AKI in PICU patients

Item	B	S.E.	Wald	*P*	*OR*	95%*CI OR*	
						upper	lower
RI	52.904	41.501	1.625	.202	9.459E22	.000	2.003E58
WBC	.575	.495	1.353	.245	1.778	.674	4.687
CRP	.045	.053	.725	.394	1.046	.943	1.159
EF	−.463	.287	2.609	.106	.629	.359	1.104
constant	−17.147	27.521	.388	.533	.000		

*PICU* Pediatric intensive care medicine, *AKI* Acute kidney injury, *RI* Renal index, *WBC* White blood cell, *CRP* C-reactive protein, *EF* Ejection fraction

This study found statistical differences in WBC and CRP between the control and study groups (*P* < 0.05). Those two indexes are both about inflammation. As stated earlier, severe infections are high-risk factors for AKI. Previous studies had found that patients with sepsis AKI associated with capillary endothelial injury, renal interstitial neutrophils, and other inflammatory cells infiltration. Simultaneously, the body of the active oxygen free radicals and reactive nitrogen free radicals produce too much will lead to oxidation and antioxidant imbalance, leading to renal tissue injuries [12]. The imbalance of the NF-κB signaling pathway is the primary mechanism of AKI, which is essentially an inflammatory change and a microcirculation disorder. There were statistical differences in inflammatory indicators between the two groups in the present study, confirming that AKI is an inflammatory response. The univariate analysis also showed a statistical difference in the length of hospital stay between the two groups. Previous research suggested that the occurrence of AKI would increase hospital stay and extend the duration of mechanical ventilation [13]. It demonstrates that the presence of AKI can significantly increase the use of medical resources and that early detection and diagnosis of AKI have important clinical implications.

In this study, renal ultrasound and NIRS monitoring were performed in patients with AKI within 24 h of admission. We found that RRI increased and RrSO2 decreased before creatinine increased, and renal blood perfusion semi-quantitative score decreased, especially in critically ill patients (see Fig. 4). As the patient’s disease improves, RRI, RrSO2, and renal perfusion change early. These findings indicated that RRI and RrSO2 changes occurred earlier than SCr, a traditional AKI diagnostic index. RRI primarily reflects the condition of renal cortex blood flow. According to existing research, at the onset of AKI, there will be a redistribution of blood flow of cortical blood flow, and the medulla is little. Simultaneously, in the early days of AKI, to protect cardiopulmonary function, renal vasoconstriction, blood supply is reduced, resulting in the appearance of RRI, and in patients with severe infection, inflammation leads to AKI progression, and renal blood flow is decreased further [14]. A study of 92 patients with shock found that the RRI of patients with shock was higher than that of patients without shock [15]. This is consistent with shock patients having a significantly higher RRI value in this study. According to Zhao Peng et al. [16], the renal resistance index can better reflect the degree of renal injury in patients with sepsis complicated by AKI.

The study found that RrSO2 decreased in the early stages of AKI but gradually increased in critically ill patients (Fig. 5) with disease recovery, implying that RrSO2 is a good predictor of AKI. There was no statistical difference between the two groups in single factor analysis. Considering that the onset of AKI was characterized by medulla hypoxia first and cortical hypoxia later than medulla [17], RrSO2 was primarily monitored in the renal cortex, and no difference was observed in the early stage of AKI (24 h). Simultaneously, changes in RrSO2 also indicate that AKI is a microcirculation disorder. Studies have found that abnormal microcirculation perfusion and oxygen metabolism disorder are two critical factors affecting renal injury in septic shock, which is of great significance for the assessment of AKI [18]. In several recent pediatric cardiac surgeries, low oxygen saturation was positively correlated with AKI. Furthermore, Ruf et al. [19] found that low renal oxygen was positively correlated with lactic acid elevation 24 h after surgery. Francesco et al. [20] found that renal oxygen reduction on the first day after birth is closely related to AKI.

ROC curve showed that the AUC of RRI predicting the occurrence of AKI within 72 h was 0.751, which was consistent with Liu’s study [21]. RrSO2 has a reasonable specificity for the diagnosis of AKI. The early prediction value of RRI combined with RrSO2 for AKI did not improve sensitivity and specificity, and the combination only increased a certain area under the curve, which was consistent with the result of the single factor analysis finding no statistical difference between RrSO2 and AKI. However, the sensitivity, specificity, and AUC of RI and their combination were higher than the current gold standard for AKI. NIRS can well reflect the oxygenation of the microvascular [22], RRI and the RI joint RrSO2 can better predict the occurrence of AKI, and can be used as an early predictor of clinical predict AKI.

Unlike previously thought, the mechanism of AKI mainly lies in glomerular and tubular cell necrosis caused by insufficient renal blood flow [23]. At present, abnormal renal microcirculation and mitochondrial dysfunction are considered to be important mechanisms for the occurrence and development of AKI [24]. RRI and RrSO2 are both non-invasive indicators. And their combination has significant implications for the early treatment of AKI, which can be applied in future studies. Xing Zhiqun et al. [25] found that high RRI (RRI > 0.7) and low urinary oxygen partial pressure (< 448 mmHg) are independent risk factors for AKI in septic shock patients, and combined application of RRI and urinary oxygen partial pressure can be an early predictor of AKI in septic shock patients.

However, this study also has the following limitations. It is a single-center, prospective study with a small sample size that must be expanded regularly. Meanwhile, the application of RrSO2 still has many limitations [26]. NIRS can only reflect the local tissue oxygen saturation and not the overall oxygen saturation of the organ. Simultaneously, NIRS measurements are influenced by height, weight, local tissue, muscle, and subcutaneous fat. As a result, many limitations exist if the children have edema, obesity, or are underweight.

## Conclusions

To summarize, AKI is a fairly common disease. For patients admitted to PICU, infection, RRI, and EF are risk factors for AKI. RRI within 24 h of admission can predict the occurrence of AKI within 72 h, RRI combined with RrSO2 can better predict the occurrence of AKI, which can be used as an early indicator for the prediction of AKI.

## Data Availability

The data and material used or analysed during the current study are available from the corresponding author on reasonable request.

## Abbreviations

AKI: Acute kidney injury
AUC: Area under curve
CI: Cardiac index
CRP: C-reactive protein
DO2: Oxygen delivery
EF: Ejection fraction
HB: Hemoglobin
LAC: Lactate
MAP: Mean arterial pressure
NIRS: Near infrared spectroscopy
PCIS: Pediatric critical illness score
PCT: Procalcitonin
PI: Pulpability index
PICU: Pediatric intensive care unit
ROC: Receiver operating characteristic
RRI: Renal resistance index
RrSO2: Renal oxygen saturation
SCr: Serum creatinine
SV: Stroke volume
SVI: Stroke volume index
SVRI: Systemic vascular resistance index
SVV: Stroke volume variability
